# Diffusion tensor imaging of frontal lobe white matter tracts in schizophrenia

**DOI:** 10.1186/1744-859X-5-19

**Published:** 2006-11-28

**Authors:** Monte S Buchsbaum, Peter Schoenknecht, Yuliya Torosjan, Randall Newmark, King-Wai Chu, Serge Mitelman, Adam M Brickman, Lina Shihabuddin, M Mehmet Haznedar, Erin A Hazlett, Shabeer Ahmed, Cheuk Tang

**Affiliations:** 1 Department of Psychiatry, Mount Sinai School of Medicine, New York, New York, USA; 2 Department of Psychiatry, University Hospital Heidelberg, Ruprecht-Karls-University, Heidelberg, Germany; 3 Taub Institute for Research on Alzheimer's Disease and the Aging Brain, College of Physicians and Surgeons. Columbia University, New York, New York, USA; 4 Department of Radiology, Mount Sinai School of Medicine, New York, New York, USA

## Abstract

We acquired diffusion tensor and structural MRI images on 103 patients with schizophrenia and 41 age-matched normal controls. The vector data was used to trace tracts from a region of interest in the anterior limb of the internal capsule to the prefrontal cortex. Patients with schizophrenia had tract paths that were significantly shorter in length from the center of internal capsule to prefrontal white matter. These tracts, the anterior thalamic radiations, are important in frontal-striatal-thalamic pathways. These results are consistent with findings of smaller size of the anterior limb of the internal capsule in patients with schizophrenia, diffusion tensor anisotropy decreases in frontal white matter in schizophrenia and hypothesized disruption of the frontal-striatal-thalamic pathway system.

## Background

The hypothesis that schizophrenia is an illness arising from disconnection or misconnections of circuits connecting prefrontal cortex with the thalamus and striatum has received support from studies of post-mortem brains [1,2], imaging [3-5], and electrophysiology[6]. The thalamus has been viewed as a key element in potential schizophrenia-related deficits in this circuit because of the reciprocal circuitry between the prefrontal lobe and the medial dorsal nucleus [7-9]. The thalamus comprises multiple nuclei that relay and filter sensory and higher order inputs to and from the cerebral cortex and limbic structures; thus, deficits are consistent with behavioral abnormalities in schizophrenia [10]. The medial dorsal nucleus (MDN) of the thalamus, important in attention as reviewed elsewhere [11], has interconnections with the dorsolateral prefrontal cortex [12]. The connections of the MDN have been used to define the prefrontal cortex [13], a key area of functional and structural alteration in schizophrenia (reviewed by: [14]. Since deficits in the frontal lobes, the thalamus, and the striatum have been found in schizophrenia with both structural and functional imaging as reviewed elsewhere [14,15], the white matter connections between the thalamus and prefrontal cortex are of special interest.

The anterior limb of the internal capsule contains fronto-thalamic and thalamo-frontal pathways, the cortico-pontine pathways, and a lesser number of caudate/pallidum fibers, while the posterior section of the anterior limb of the internal capsule has primarily corticopontine fibers [16] Reduction in the size of the internal capsules in patients with schizophrenia [17-20] would be consistent with diminished corticothalamic and corticostriatal connectivity and with previous reports of schizophrenia-associated abnormalities in the striatum [21,22] and thalamus [4,5,23,24].

To investigate white matter circuitry of these frontal-thalamic connections, we applied diffusion tensor magnetic resonance imaging (MRI) to a small group of patients with schizophrenia (*n *= 5) and found reduced organization of white matter, as inferred from anisotropy, in the frontal lobe and anterior limb of the internal capsule [25], a finding replicated by Lim and collaborators [26]. Frontal decreases in anisotropy in schizophrenia have been confirmed and extended in other reports [27-32]. In some reports, frontal lobe and fronto-temporal tracts including the arcuate fasciculus [33], anterior cingulum bundle [34,35], uncinate fasciculus [36,37] and superior longitudinal fasciculus [36] were found to show low anisotropy; by contrast, no white matter area emerged as significantly low in studies of 14 [38] and 10 schizophrenic patients [39].

An alternative viewpoint to the possibility of misrouting or specific tract deficits in white matter in schizophrenia is a global deficit in myelin [40] (and see review [41]). This might produce a pattern of distributed multiregional anisotropy deficits compatible with the complex, not clearly localizing, behavioral and cognitive disorganization in schizophrenia. Alteration in number, distribution, and ultrastructural integrity of oligodendrocytes, key white matter components, has recently been reported in the prefrontal cortex in schizophrenia [42-45].

One approach to differentiating a global myelin deficit from specific tract deficits is to examine statistical parametric mapping, which has tended to show deficits in frontal white matter, as reviewed above. This issue can also be approached by tracing the path of diffusion from voxel to voxel, termed tract tracing, to make inferences about the course of axon bundles. The fronto-thalamic connections can be explored by tracing tracts between the anterior limb of the internal capsule forward to the prefrontal cortex.

Anisotropy analysis is limited to a single number expressing the directionality of water diffusion at a single voxel with high values indicating all diffusion in one direction and low values indicating diffusion in all directions. Diffusion tensor analysis also yields the 3D vector expressed as the component of the x, y, and z dimensions of the diffusion. The ending point of a tract (the xyz location) and length of the tract are estimated [46]. This method proceeds from a starting voxel and locates an adjacent voxel as part of the tract if the anisotropy exceeds a preset threshold and if the solid angle of the vector is no greater than 30 degrees. Thus a tract can be traced from an outlined area in the internal capsule moving toward the prefrontal cortex. If myelin deficits are general, tract tracing would terminate in a shorter distance from the starting voxel than expected. However, if the tract terminated in a different position or had a different length, then a miswiring deficit would be supported. Thus, if we hypothesize a general myelin deficit, short tracts might be widespread. Alternatively, we might hypothesize patients with schizophrenia to have more poorly organized fibers, fanning out to the cortex irregularly with less topographic precision and perhaps earlier in their course. Such patterns have been observed in cortical dysplasia in elegant single case diffusion tensor tract tracing examples [47]. Their illustrations suggested that tract length might be a useful measure in examining deficits in the cortex in schizophrenia.

## Methods

### Subjects

The schizophrenia group consisted of 103 patients (83 men, 20 women) recruited from inpatient, outpatient, day treatment and vocational rehabilitation services at Mount Sinai Hospital (New York, N.Y.), Pilgrim Psychiatric Center (W. Brentwood, N.Y.), Bronx VA Medical Center (Bronx, N.Y.), Hudson Valley Veterans Affairs Medical Center (Montrose, N.Y.), and Queens Hospital Center (Jamaica, N.Y.) following approvals by each institutional review board and informed consent obtained from each subject. The 41 matched normal control subjects (28 men, 13 women) were recruited through advertisement. All schizophrenia patients met DSM-IV diagnostic criteria for schizophrenia (n = 92) or schizoaffective disorder (n = 11). Diagnosis was determined by a structured clinical interview with the Comprehensive Assessment of Symptoms and History (CASH; [48]. Patients were moderately ill (positive and negative syndrome scale scores (PANSS; [49] for positive, negative and total were 18.9 ± 6.6, range 8–38; 18.9 ± 7.8, range 0–41; 37.0 ± 9.9, range 19–73 respectively, obtained on n = 97). The Mini-Mental Status Examination scores were 26.9 ± 2.7, range 16–30, obtained on n = 91). Patients were for the first time administered neuroleptics as ascertained by medical records by the age of 24.9 ± 9.1, indicating that the average patient had received some treatment over a period of 19 years. At the time of the scan we examined records covering the preceding three years and identified the predominant treatment: 11 were unmedicated, 20 were on conventional neuroleptics, 37 on atypical neuroleptics, 16 on both, and the remainder were on other psychoactive medications in addition to neuroleptics or the history of the last period was poorly documented.

Schizophrenia patients were divided into good-outcome (non-Kraepelinian, n = 51) and poor-outcome (Kraepelinian, n = 52) subgroups based on published criteria [50]. Poor-outcome patients met the following criteria for at least the five years prior to study contact: 1) continuous hospitalization, or, if living outside the hospital, complete dependence on others for food, clothing, and shelter; 2) no useful work or employment; and 3) no evidence of symptom remission. All other schizophrenia patients were considered good-outcome, or non-Kraepelinian.

There was no significant difference in age between schizophrenia patients (mean age 43.0 ± 12.4) and normal controls (mean age 44.1 ± 14.7) (t (142) = -0.47, p = 0.64) and sex distribution (χ^2^(1) = 1.86, p = 0.17). MRI volumetric data from participants in the current study have previously been reported in studies of the thalamus, striatum, and Brodmann areas of the cortex [51-54].

### Image acquisition

The diffusion tensor sequence acquired fourteen axial 7.5-mm-thick slices (TR = 10 s, TE = 99 ms, TI = 2.2 s, b = 750 s/mm, δ = 31 ms, Δ = 73 ms, NEX = 5, voxel 1.8 × 1.8 × 7.5 mm, FOV = 230 mm) and the SPGR (spoiled gradient recalled acquisition in steady state) anatomical sequence acquired 124 1.2-mm-thick slices (TR = 24 ms, echo time = 5 ms, flip angle = 40°). This sequence was chosen because of its data-acquisition speed, an important consideration when imaging schizophrenia patients. It also allows us to perform signal averaging to improve the signal-to-noise ratio (SNR). Before the diffusion EPI sequence, a Turbo Spin Echo (TSE) is also acquired to obtain a localizing anatomical image.

To assess the degree of diffusion anisotropy in each voxel, we used fractional anisotropy (FA). This quantity is a measure of the degree of anisotropy in a voxel, the degree to which the diffusivity is biased along the fiber axis as opposed to perpendicular to it. In order to solve for the components of the diffusion tensor, seven diffusion EPI images were then obtained: six with different non-collinear gradient weightings and one with no diffusion gradient applied. Five acquisitions were then obtained and averaged to improve the SNR. The diffusion tensor was then obtained by solving the seven simultaneous signal equations relating the measured signal intensity to the diffusion tensor. We then obtained a tensor for every voxel in a slice. We then computed the eigenvectors and eigenvalues for every tensor, which form the basic raw data set that was analyzed subsequently. The eigenvector associated with the largest eigenvalue or principal diffusivity indicates the direction along which the apparent diffusivity is at a maximum, which in normal white matter corresponds to the orientation of the axis of an axonal bundle, and allowed us to obtain information about the orientation of maximum diffusion, which in normal white matter would correspond to the axis of an axonal bundle.

We also determined that images were processed with the averaging for 5 NEX done in the Matlab routine for the first subjects and using GE software for the later subjects. Although visual inspection of the anisotropy images revealed no apparent differences in the earlier or later scans, statistical analysis of whole brain white matter FA showed small but significant differences between the two sets of data. We are not specifically able to identify the sources of variation. No subjects were scanned with both variants of the method. Therefore we first corrected for potential variation over time by dividing by the whole-brain average fractional anisotropy. This is quite analogous to correcting BOLD or FDG PET values to whole brain activity levels as is widely done and also controls unwanted drift effects over time.

Anatomical MRIs were resectioned to standard Talairach-Tournoux position using the algorithm of Woods et al. [55] and a 6-parameter rigid-body transformation. The anisotropy images from each subject were then aligned to subject's own standard-position anatomical images using the same 6-parameter rigid-body transformation. Note that both the structural and diffusion tensor images remained in their original dimensions and were only aligned using the 6-parameter alignment.

We tested the extent of error of coregistration and potential distortion of the diffusion tensor images from the less distorted structural MRI and the diffusion tensor anisotropy images as described in detail elsewhere [56]. The median difference of the absolute image frame coordinates of the anterior and posterior brain edges between structural and anisotropy images was 0.0 and 1.78 mm respectively and the median difference in brain length was 2.23 mm, just above 1%. The mean absolute value in mm of the differences between the diffusion tensor and MRI locations in the anterior and posterior brain edges was +2.14 and +2.71 mm respectively. No difference between images collected with the different nex averaging methods was statistically significant.

### Tract tracing

We followed the technique of [46] beginning with every pixel in the internal capsule at each slice level. The criterion for continuing the path was a relative anisotropy value of 0.7, interpolated steps of 0.3 mm and a change of less than 30 degrees from voxel to voxel. Note that this is a value relative to whole brain. Each tract length is measured from the individual voxel location in the internal capsule region of interest along the tract to its termination.

### Manual tracing of the anterior limb of the internal capsules

The left and right anterior internal capsules were manually traced in the axial plane on five dorsal-to-ventral equidistant levels [18]. The five slices were determined based on anatomy of the striatum, following Buchsbaum and colleagues [52]. Briefly, the most ventral and most dorsal slices containing the putamen were selected. The most ventral aspect was defined as the first slice in which the caudate and putamen were no longer merged. The most dorsal slice was defined as the first slice in which the putamen was no longer visible. The number of slices between the most dorsal and most ventral slices was divided by 6 to yield an increment accurate to two decimal places. This increment was added to the bottom slice number five times and the result rounded to yield five equally and proportionately spaced slices for tracing. The internal capsule was traced on the five slices corresponding to the five equidistant spaced slices of putamen.

**Figure 1:**
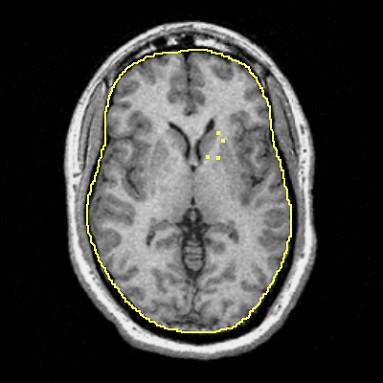
Axial MRI with four corner markers of the internal capsule placed.

The tracing protocol was developed after consultation with a neuroanatomist, expert in cerebral white matter, with the intent of maximizing consistent measurements across subjects while capturing fibers restricted to the anterior limb of the internal capsule region. Four manually-inserted landmarks were used to create the "corners" of a polygon containing the fibers of the internal capsule. For each hemisphere, a landmark was placed on the most lateral anterior part of the caudate nucleus to define the anterior medial corner of the internal capsules. A landmark was placed on the most medial anterior part of the putamen to define the anterior lateral corner of the internal capsule. For the more posterior medial and lateral corners, a landmark was placed on the most posterior part of the caudate nucleus, and one was placed on the most medial aspect of the putamen, respectively. An automatic boundary-finding method, based on the Sobel-gradient filter allowed for maximization of grey/white matter contrast for accurate placement of landmarks on the borders between the striatum and internal capsules. Figure 1 displays an example of placement of landmarks on the five equidistant slices. To determine interrater reliability, ten subjects were chosen randomly and traced by two independent operators and total volumes, across hemisphere, were compared between the two. The intraclass correlation (ICC) was 0.74, which is consistent with other studies that have examined the tracer reliability of small structures. An automatic computer program developed by MSB was used to compute the area of the internal capsule at each slice, which was contained in a polygon formed by the four landmarks.

In a second step, we located the anterior corpus callosum on an axial slice at the dorsoventral level of the third out of five internal capsule levels (vertical center) and determined the brain midline (x) and most anterior border of corpus callosum white matter (y). We used this position to further standardize the xy coordinates of the anterior limb of the internal capsule and termination of tracts in the frontal lobe as it was symmetrically in the midline and determined in exact reference to our traced volume of the internal capsule. This reduced the variation which was introduced by using the Woods algorithm brain standardization which yielded some residual dorsoventral variation at this level.

We examined both raw tract length and tract length adjusted for brain size. Since the major tract length differences were in the axial plane, we first determined the midline of each axial slice, defined as 90 degrees. We then determined the angle from the midline formed by the line joining the centroid of the internal capsule with the centroid of the tract end and the length and width of the brain. We divided the tract length by the brain length multiplied by a correction for brain proportion (the angle between the midline and the tract angle divided by 90 ; e.g. if the brain length was 200 and the brain width 100 and the angle of the tract end 45 degrees from the midline, then the correction factor for brain size was 200 minus ((200 minus 100) × ((90–45)/90)) or 200 minus 50 which is 150. We then divided the tract length by 150, the measure of axial brain size.

## Results

### Tract length

**Figure 4:**
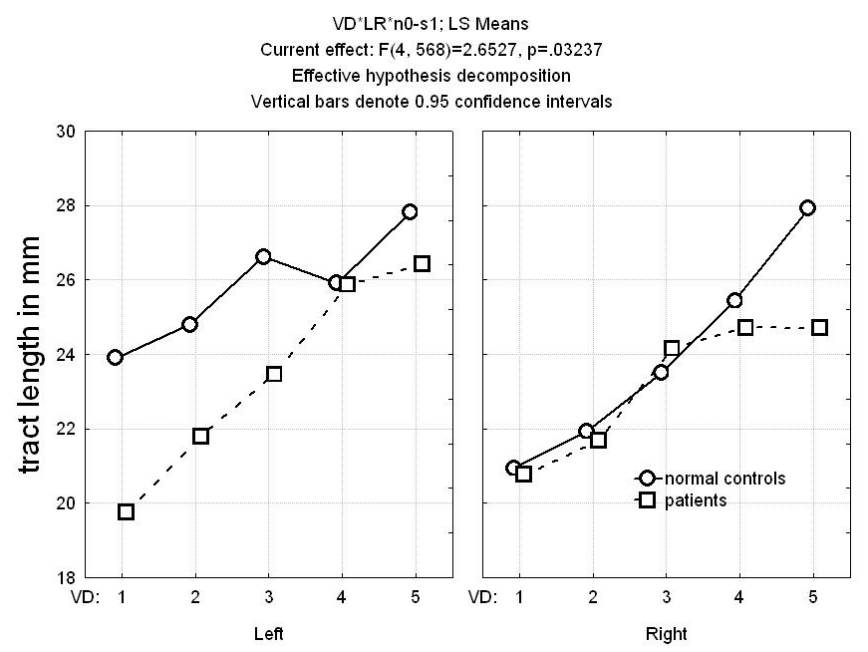
Mean tract length at five ventrodorsal internal capsule positions for normal controls and patients with schizophrenia. In the left hemisphere, the patients' tracts were shorter than normal volunteers at all five levels but the difference was most marked at the ventralmost levels. For the right hemisphere the difference was present only for the most dorsal two levels (group × ventrodorsal level × hemisphere interaction, F = 2.65, df = 4,568, p = 0.032; Wilks 0.92, F = 3.01, df = 4,139, p = 0.02).

Average tract path lengths in mm from the internal capsule voxel to the tract termination centroids entered into in a four-way ANOVA with group as an independent measure and length, ventrodorsal level, and hemisphere as repeated measures revealed that in the left hemisphere patients with schizophrenia revealed shorter tracts in all ventrodorsal levels and in four of five right hemisphere ventrodorsal levels (Figure 4), and differences were greatest in the left hemisphere at ventral levels (group × ventrodorsal level × hemisphere F = 2.65, df = 4,568, p = 0.032; MANOVA Wilks 0.92, F = 3.01, df = 4,139, p = 0.020; corrected for brain size: group × ventrodorsal level × hemisphere F = 2.53, df = 4,568, p = 0.040; multivariate F = 2.79, df = 4,139, p = 0.029). Post-hoc t-tests were not significant.

**Figure 5:**
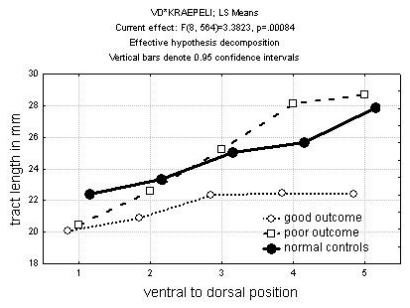
Tract length in good and poor outcome schizophrenia. Note that both good and poor outcome patients have shorter tracts at the ventralmost level.

Both good and poor outcome patients had shorter tract lengths than normal controls at ventral levels, but good outcome patients had shorter tracts than normal controls or poor outcome patients at dorsal levels; they lacked the normal ventrodorsal gradient (group × ventrodorsal level × hemisphere interaction F = 3.38, df = 8,564, p = 0.0084) (Figure 5).

### Anisotropy

Patients with schizophrenia had lower anisotropy within the tracts ventrally but not dorsally compared to the controls (group × ventrodorsal level × hemisphere interaction F = 3.20, df = 4,568, p = 0.013; but multivariate F 2.31, p = 0.061) Although patients and normals were well matched for age, we carried out ANCOVA to remove age effects and the result remained significant (F = 3.14, df = 4, 564, p = 0.014).

### Tract path termination locations

We separately examined the x, y, and z coordinates of the centroids of tracts termination positions, for the tracts in each subject that originated anterior limb of the internal capsule. Three-way ANOVAs separately performed on x and z directions, with diagnosis as an independent group dimension and dorsoventral level and hemisphere as repeated measures revealed no significant main effects or group interactions. An ANOVA performed on the y-coordinate of the centroid of tracts' terminations revealed that in the left hemisphere the tracts of normal controls terminated more anteriorly than the tracts of patients with schizophrenia for all ventrodorsal levels except level 4, where there was no difference between the normals and the patients (1 = most ventral, 5 = most dorsal). In the right hemisphere, the tracts terminations are practically the same for the ventral levels 1 and 2 in normals and schizophrenics, but in level 3 the schizophrenics are more anterior in their tract termination than the controls. For the dorsal levels 4 and 5 in the right hemisphere, the pattern resembles the left hemisphere with the normals having more anterior points of tract termination as indicated by the more anterior y-coordinate of their centroids (group × ventrodorsal level × hemisphere interaction, F = 2.725, df = 4,568, p = .029; Wilks 0.92, F = 2.97, df = 4,139, p = 0.022).

### Internal capsule size and tract length

In normals, there were no significant correlations between size of the internal capsule and tract length at any of the five levels in either hemisphere. For patients, only the left most ventral level showed a correlation (r = -0.21) indicating that smaller internal capsules were associated with greater length. Thus, internal capsule size does not appear to be a major determinant of tract length.

### Number of starting locations for tracts

The number of starting locations at the ventralmost internal capsule (left and right combined for normal controls (151 ± 22) and patients (147 ± 28) did not differ (t = -.72, df = 142, p = 0.47). Only the dorsalmost level differed with normals (227 ± 45) higher than patients (209 ± 43) differed significantly (t = -2.26, df = 1,142, p = 0.026).

## Discussion

**Figure 6:**
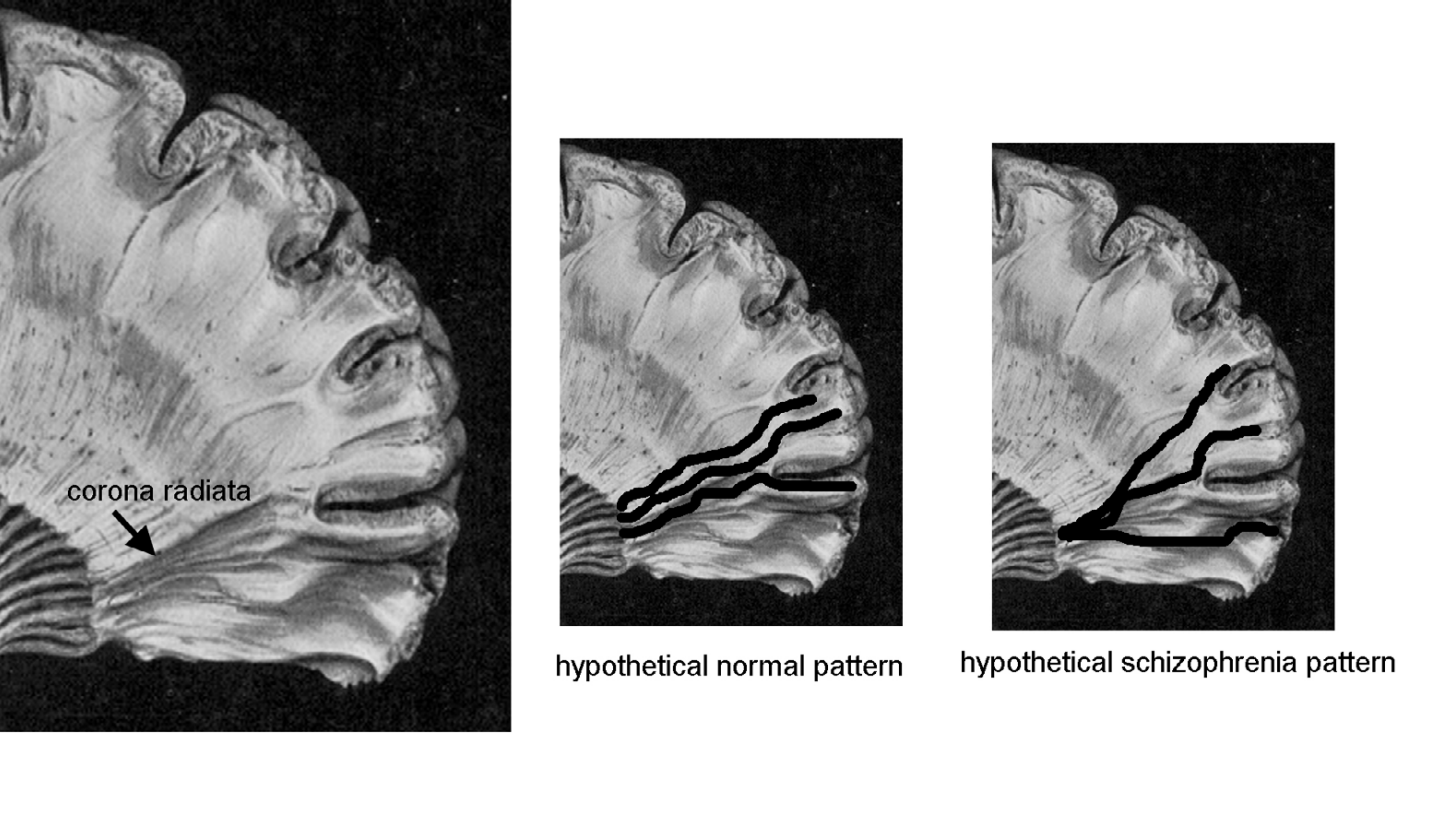
Hypothetical arrangements of corona radiate fibers in normals and patients with schizophrenia. In the normal pattern, tract length would be longer than in the more radial divergence seen in patients with schizophrenia.

The finding of shorter tract lengths between the anterior limb of the internal capsule and the prefrontal cortex is consistent with an abnormality in thalamo-cortical and cortico-thalamic connectivity, with findings of reduced size of the anterior limb of the internal capsule in schizophrenia, postmortem and MRI findings of smaller medial dorsal nucleus volume in schizophrenia and with the perceptual and cognitive abnormalities in schizophrenia. Shorter tract lengths might result from a less fully topographically developed fiber tracts (Figure 6).

The findings are also consistent with the appearance of anisotropy images which show a very marked line running from the internal capsule in an anterior direction toward the frontal pole. The significant difference in the length of the tracts is primarily due to the difference in the y-component of the distance from the centroid of the internal capsule to the tract termination, and not to either x or z components. We note that both reported ANOVA's on the y component and on the three-dimensional distance in mm reveal similar F-ratio values, indicating the effect is due to y-dimension (anterior-posterior axis).

The shorter tract length in patients does not seem to be attributable to a different number of tract-tracing starting positions, since the biggest differences in tract length between normals and patients are at the ventralmost levels where the number of intial tract starting positions within the internal capsule are the same in both groups.

### Validity of tract tracing, potential confounders, and study limitations

We oriented the tracing of the vectors from the internal capsule toward the prefrontal cortex to specifically evaluate thalamo-frontal and fronto-thalamic fibers. Note that the diffusion tensor data does not differentiate between thalamo-frontal and fronto-thalamic directions, but only yields the orientation of the water diffusion.

Validity of the tract-tracing algorithm is supported by the systematic topographic organization of the tract termination location and, as noted above, the marked visibility of anteroposterior white markings in the y-direction images of anisotropy [46]. However the tract tracing is limited by the resolution of the diffusion tensor images and the relatively conservative threshold (0.7) used to terminate tracts. The presence of tracts crossing the anteroposterior course of the thalamocortical fibers, differentially increased head motion in patients with schizophrenia, frontal distortion of diffusion tensor images, and errors in MRI coregistration all affect the accuracy of the results. We may also have less power to find z-axis effects since the within axial plane (xy) resolution is greater than the z resolution.

Shorter tracts were not clearly related to lower tract anisotropy, since only one of the ten tract tracings (5 levels in two hemispheres) showed significantly lower anisotropy in the tract itself. Our region of interest was not defined by tract orientation, so it contained a mixture of frontothalamic, frontopontine and caudatopallidal fibers[16]. While our method of actually outlining the edges of a white matter area gives it some specificity, Kanaan et al [57] point out the advantages of adding tract direction information to determining the roi edge. Note that FA has been found to be reliable across sections [58]

### Functional neuroanatomy and schizophrenia function

The tract lengths terminate before reaching the cortical rim. This may result from the fibers fanning out to reach a sector of the cortex earlier in their course (Figure 6) and may suggest a different geometry of prefrontal connectivity. It may also represent fibers which have a tortuous course and are therefore interpreted as terminated by the 30 degree criterion or the presence of right to left fibers crossing the anterior-going paths. Changes in internal capsule size due to ventricular enlargement might also affect the tract length, perhaps especially within the internal capsule. More detailed analysis of the angles of fibers at the anterior edge of the internal capsule and within the internal capsule, and correlations with ventricular size, prefrontal and thalamic volumes, and cortical thickness may be informative in extending our understanding of this finding.

### Summary

The length of tracts from the anterior limb of the internal capsule to the prefrontal cortex was found to be shorter in patients with schizophrenia than age- and sex-matched normal controls. This suggests a deficit in connectivity between the association nuclei of the thalamus, especially the medial dorsal nucleus, and the executive areas of the prefrontal cortex. Such a deficit might be associated with the degraded performance on attentional and perceptual tasks widely observed in schizophrenia.

**Figure 2:**
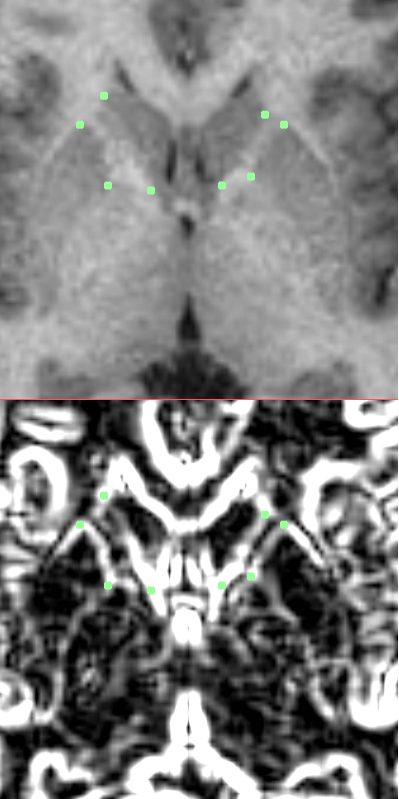
Top: Enlarged view of internal capsule with four corners marked. Bottom: Sobel gradient filtered image with enhanced edges of caudate and putamen marked.

**Figure 3:**
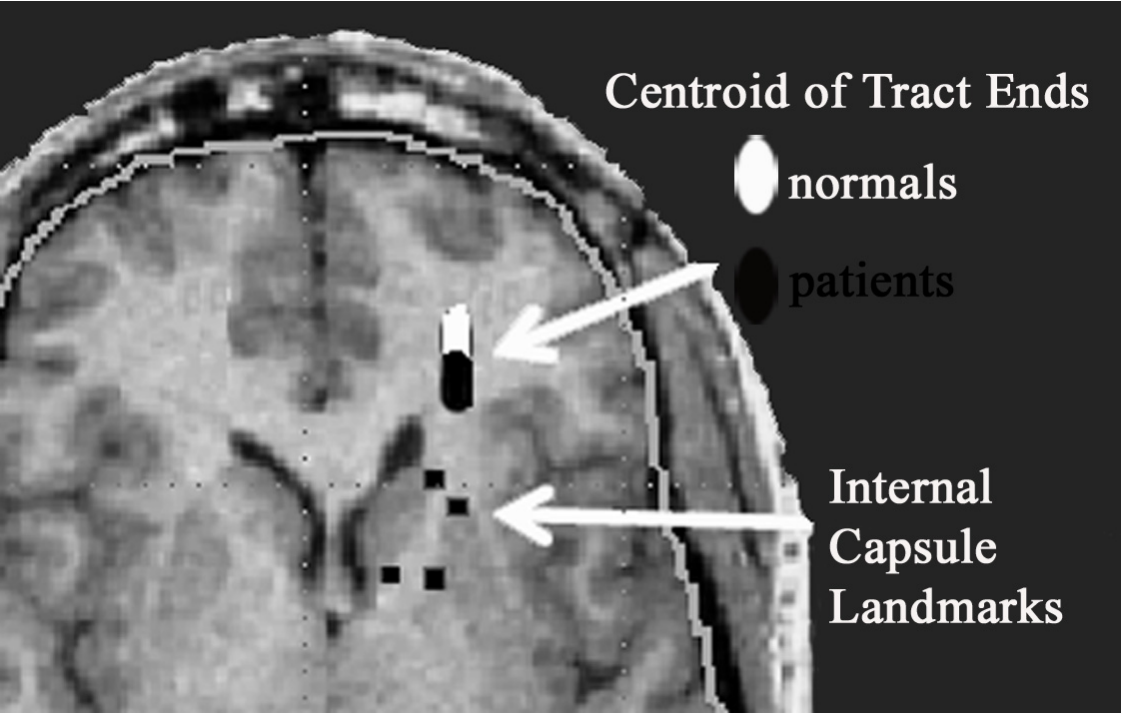
Location of average tract endings. White oval has center placed at mean xyz location of the centroid of each tract ending location across all normal subjects and the width and length of the ellipse are equal to one standard deviation of the x and y coordinates. The black oval portrays the same data for patients with schizophrenia
